# Evaluation of two commercial and three home-made fixatives for the substitution of formalin: a formaldehyde–free laboratory is possible

**DOI:** 10.1186/1476-069X-11-59

**Published:** 2012-09-04

**Authors:** Cristina Zanini, Elisa Gerbaudo, Elisabetta Ercole, Anna Vendramin, Marco Forni

**Affiliations:** 1 Research Laboratory of EuroClone S.p.A at Molecular Biotechnology Centre (MBC), University of Turin, Turin, Italy; 2 Department of ImmunoHematology, A.O. Ospedale Infantile Regina Margherita (OIRM), S.Anna, Turin, Italy; 3 Research Laboratory of EuroClone S.p.A, Basovizza, TS, Italy

**Keywords:** Formalin toxicity, Alternative fixatives, Tissue micro array, RNA extraction

## Abstract

**Background:**

Formaldehyde (HCHO) is a gas (available as a 37% concentrated solution, stabilized with methanol). The 10% dilution (approximately 4% formaldehyde) has been used as a fixative since the end of the 19th century. Alternative fixatives are also commercially available or may be prepared in-house in laboratories. Statements by the IARC, along with other USA agencies (CalEPA, RoC/NTP) on the carcinogenicity of formaldehyde for humans renders its substitution in Pathology Departments necessary since the annual use of formalin may exceed 3,500 liters for a medium-large laboratory.

To achieve a “formalin-free laboratory” we tested straightforward-to-make fixatives along with registered reagents offered as formalin substitutes.

**Methods:**

More than two hundreds specimens were fixed in parallel with in-laboratory made fixatives PAGA (Polyethylenglycol, ethyl Alcohol, Glycerol, Acetic acid), two zinc-based fixatives (ZBF, Z7), and commercially-available alternatives (RCL2 and CellBlock). Tissue micro arrays were used for morphological and immunohistochemical comparison. Extraction of RNA was carried out to evaluate preservation of nucleic acids.

**Results:**

Differences compared to formalin fixation were evident in alcohol-based fixatives, mainly restricted to higher stain affinity and considerable tissue shrinkage. Conversely, nuclear detail was superior with these alcohol-based formulas compared to formalin or glyoxale-based recipes. RNA extraction was superior for Z7, PAGA and RCL2 with regard to concentration but relatively comparable regarding quality.

**Conclusions:**

Abolition of the human carcinogen formaldehyde from pathology laboratories is possible even in contexts whereby commercial alternatives to formalin are unavailable or are too expensive for routine use, and aspiration devices are lacking or not adequately serviced. The use of known formulations, possibly with simple and not-noxious (“alimentary grade”) constituents, comparable with registered proprietary products, may expand the search for the ideal fixative combining satisfactory morphology with improved preservation of nucleic acids and proteins as well as being easy and safe to dispose of.

## Background

Fixation is a key step in the practice of diagnostic pathology, and even in modern times, is intimately linked to the use of formaldehyde (HCHO), a gas commercially available in concentrated solution (37%), stabilized with ethanol, and named Formalin. A 10% dilution of concentrated formalin (in tap water or buffers with final concentrations of formaldehyde of approximately 4%) is named 10% formalin [1,2].

The main motives for the popularity of formalin among pathologists are: low cost, straightforward laboratory preparation procedure, long-standing tradition and international use.

There is a general consensus that formalin is the best fixative and there is therefore no need for improvement for any reason, generating what has been defined by some authors as “the formalin dogma” [3], a somewhat fatalistic and “addictive” approach that has severely hampered the search for alternatives to formalin in fixation procedures.

Political changes in recent decades, along with the generalized shift towards a “market-regulated economy”, have introduced economy-driven research, and the proprietary use of innovative and scientific observations, along with a more open approach as well as the free exchange of scientific knowledge. However, the free marketing of alternative fixatives with registered and proprietary formulas have in fact acted as an obstacle to the diffusion and sustained use of registered products [4]. It has also discouraged the quest and testing of formalin substitutes for every-day use, and the widespread scientific research and investigation into new fixatives.

The toxicity of formaldehyde is emerging as the main issue for its abolition as a general fixative used in large quantities in pathology laboratories (a medium-large structure may annually use more than 3,500 liters of ready-to-use formalin).

In 1987, the U.S. Environmental Protection Agency (EPA) classified formaldehyde as a probable human carcinogen under conditions of unusually high or prolonged exposure [5] and a few years later the permissible exposure level was lowered from 1 ppm to 0.75 ppm (average daily exposure) [6] and the potential toxicity of formaldehyde exposure to technicians and pathologists was stressed, meanwhile promoting the search for alternative fixatives.

More recently, from the initial IARC report linking formaldehyde exposure to human nasopharyngeal carcinoma [7], a successive link between formaldehyde exposure and leukemia was put forward in 2009 [8]; these statements were reinforced in the 2012 report [9] by the same agency. These data of the carcinogenicity for humans of formaldehyde should act as a potent stimulus to reconsider the “formalin dogma” and to evaluate with an open mind the use of alternative fixatives.

The U.S. Occupational Safety and Health Administration (OSHA) stated that employers must reduce worker exposure to formaldehyde at, or below, permissible exposure limits (PEL) and the TWA (time-weighted average) should be less than or equal to 0.75 ppm. The 15-min short term exposure limit (STEL) is 2 ppm. [10].

International and National agencies set stringent limits of formaldehyde exposure ranging from 0.016 ppm TWA (National Institute for Occupational Safety and Health, USA) to 2 ppm (OSHA and Australia) for STEL [11].

Moreover, the formation of DNA-Protein cross-links (DPC or DPX) possibly represents a permanent “signature” of exposition to formalin [12]. More recently chromosomal alterations have been detected in health workers and linked to formalin use in Pathology Wards [13]. This information was largely ignored by the pathologist community who assumed that the use of formaldehyde as a fixative is mandatory and thus completely safe when used with adequate procedures [14].

Recent reports comparing a group of pathologists and industrial workers, and using individual devices to monitor exposure to formaldehyde demonstrated that environmental exposure in pathology departments is trivial in most areas while the sampling activity in the gross room may result in exposure to toxic levels that exceed the recommended values [15,16]. Individual exposure to formaldehyde was monitored by measuring Malondialdehyde-deoxyguanosine adducts on peripheral leukocytes and the alkylation of hemoglobin to form a terminal N-methylene valine residue. These data show that formaldehyde exposure in the gross room is comparable to that of workers in plastic factories and that perfectly efficient aspiration devices may not in fact avoid individual exposure.

Nevertheless, law restrictions on the use of human carcinogenic substances, mainly directed at industrial production and use, are also valid for pathology laboratories. In Italy, safety labels on formaldehyde preparations have not changed from irritant (X) to carcinogenic substance (skull pictogram). One of the aims of our study is to clarify the necessity of general awareness of the scientific and ethical issues related to the abolition of formaldehyde use in pathology laboratories, and to contribute to the quest for valid alternatives, either homemade or commercially-available.

On the other hand, this classification into a Class I substance makes surveillance of exposed personal mandatory, and may involve Directors of Surgical Pathology Departments as well as Hospital Administrators responsible for the health of workers. The delay in introducing this new information as a National Standard has led to the generation of the idea that the IARC warning was limited to industrial environments and not mandatory for pathology laboratories.

However, a recent European legislation (e.g. in Italy n° 81/2008) clearly states that carcinogens should only be used in closed work-cycles and must be substituted when technically possible (Art. 235). European Union legislation is moving in the same direction.

While industries (e.g. wood and furniture) are rapidly adhering to law prescriptions (and are certifying the absence of formaldehyde vapors), in the medical field an unusual delay is still present.

A rational, open-minded and innovative approach to fixation, with the scope of abolishing formalin use altogether, [17,18] or at least limiting its use to very rare situations in which there are no available alternatives (like Formaldehyde-induced fluorescence for Catelcholamines or formaldehyde-vapor fixation in certain histo-enzymatic determinations [1]).

The use of formalin in surgical pathology laboratories may be partially justified by the large size of the surgical specimen, putatively easier to handle after formalin fixation and supposedly better fixed in comparison to alternative fixatives. However, it must be stressed that manipulation of large resection materials, along with increasing demands of more detailed macroscopical descriptions and extended sampling (as required by modern staging of tumors) may increase the exposure of pathologists to formaldehyde vapors.

However, when studying experimental animals of small dimensions no rational explanation may be proposed for the continuation of the use of formalin-fixed tissue for microscopical evaluation.

Nevertheless, the National Toxicology Program of the U.S. – probably the largest program in this field - requires the use of buffered formalin [19]. Conversely, an European Institution (Cesare Maltoni Cancer Research Center, European Ramazzini Foundation of Oncology and Environmental Sciences, Bologna, Italy. crcfr@ramazzini.it) suggests alcoholic fixation of all organs of rats apart from bones [20]. Curiously, for de-calcification the same protocol suggest a solution containing formaldehyde and formic acid, despite there being several formaldehyde-free methods of decalcification available, as reported in literature and on the market.

As these formaldehyde-free procedures are effective in human pathology for large bony samples, there is no logical reason for the continued use of formaldehyde - formic acid formulations for decalcification of bones of much smaller experimental animals.

A second more recent, but preeminent issue, is the search for a more effective way of fixing tissues in order to preserve nucleic acids and proteins for molecular biology techniques [21-25], with the perspective of utilization of the vast surgical pathology archives of tissues, for scientific research on tumors or other relevant human diseases, and to integrate these determinations into clinical diagnoses.

The importance of good preservation of tissues, not only for microscopic evaluation, but also for Histochemistry, Immunohistochemistry and Molecular Biology is taken into consideration [17,21,26,27]. The fatalistic approach is that that once a specimen is dropped in formalin, no other manipulations or control procedures are necessary. This is the case even for very large specimens that will never be adequately fixed in their central parts unless proper slicing (or alternative maneuvers) is applied. It is also given a low profile which underestimates the potential of new tools (like nucleic acid technology or proteomics) in the diagnostic process.

As highlighted in a study on the influence of fixation and processing parameters [28], the final fixation of many tissue of large specimens primarily fixed with formalin is frequently achieved during processing and is actually an alcoholic fixation. This note - contrary to efforts to standardize formaldehyde fixation and processing [29] - is a further argument against the “formalin dogma”, but it also an important rational explanation of the fact that paraffin-embedded tissues are very stable and that tissue reactivity is influenced by primary fixative, but is stabilized by alcoholic dehydration and clearing before embedding. For instance, the deleterious action of Picric Acid (a constituent of Bouin's Fixative) mainly affects DNA bases and is not related to improper fixation.

Is worth noting that a practical application of the “formalin dogma”, the acronym FFPE (formalin- fixed paraffin embedded) enforced the dogma than no other fixative but formalin should be used [29].

The use of microwaves may be a interesting way for obtaining a better fixation either as a step in fixation–processing procedures, or as an upgrade of automatic processing instruments.

Commercially-available alternatives to formalin are fairly numerous and in different supply formats either ready-for-use or in concentrated forms. While ready-to–use formulas are very convenient, concentrated formulas may allow some degree of manipulation and custom tailored modifications.

As noted by Kiernan [1] the use of “secret mixtures”, that is- the registered proprietary formulas of poorly detailed components- is a severe limitation to a rigorous scientific approach to fixation, and despite being expensive, may be a straightforward solution, at least in the short term.

Less numerous are the non- proprietary formulas proposed in recent scientific literature while the wealth of information on time-honored fixatives reported in scientific journals and histochemistry text books may be worth revisiting as they give useful clues for new improvements. The use of Zinc salt-based fixatives as alternatives to formalin was recently reported by a Swedish group [30] and subsequently upgraded by other scientists [31].

The availability of commercial alternatives to formalin is mainly restricted to Western countries, being widespread in North America, and to a lesser degree in Europe. The possibility of preparing effective substitutes in the laboratory may be of particular interest, considering the high cost of an effective and durable aspiration chain of formalin vapors (from aspiration hoods, aspirated cabinets and final proper disposal), and the limited economical resources of developing countries that frequently lack proper servicing of instruments [32].

The low cost of formalin, being less expensive than brand substitute products, and also being readily available in the market by many suppliers and in different convenient formats has hampered the diffusion and research of alternatives. Indirect costs of formalin fixation (for example: adequate disposal of used fixative and fixed specimens; potential toxicity of products from incineration of residual specimens; and the imperative necessity of perfect maintenance of aspirations devices, such as hoods and aspirated cabinets) should also be considered in economical evaluation of costs. Moreover the necessity of adequate health surveillance of workers exposed to a human carcinogen should be also considered as an additional and non-trivial economical cost.

Recently, new devices were proposed by industrial firms to avoid the exposure to formaldehyde in pathology laboratories during grossing activities [33,34] however a more radical approach for completely abolishing formaldehyde and for obligatory use of alternative fixatives not based on gas in solution seems more appropriate.

In the present study we started from the standard practice of our pathology laboratory at the Ospedale Infantile Regina Margherita (OIRM) disestablished in 2009, in which formalin use as a fixative was limited to autopsy practice, a small activity in comparison to histology and cytology.

The decision to substitute formalin was the only available possibility to protect lab personnel from gas exposure when faced with the refusal of the hospital administration board to upgrade the aspiration hoods in a reasonable time.

From 1992, formalin was substituted with a commercial formula distributed in Italy as “Biolina” but corresponding to “Histochoice” (Amresco, OH, USA). This glyoxale-based fixative with ethyl alcohol (20-40% in the ready-to-use fixative) gave an acceptable morphology in our hands, and was used for almost 3 years as a routine fixative. In the gradual process of formalin substitution, 0.5 ml of methylene blue was added to a liter of Biolina, obtaining a light blue color. This simple method permitted detection, at a glance, of the presence of the substitute fixative, and avoided the use of smelling when in doubt. The use of this “light blue fixative” was widely accepted, especially in surgical theaters. In the autopsy practice, Biolina was unsatisfactory due to a slower tissue penetration and the lack of tissue hardening that made further trimming difficult. Therefore, Formalin use was limited to post-mortem examinations with cautious procedures to limit vapor diffusion.

In order to protect the proprietary formula, the safety sheet did not fulfill the European standards on safety instructions for chemical products for laboratory or industrial use (16 points are required to be clearly stated) [35]. Subsequently Biolina was retracted from commercial use in Italy, and all known deposits of the reagent were sequestrated.

Abruptly, it was necessary to face a difficult situation in which no commercial alternative was available in the national market, and the temptation to return to formalin was therefore very high.

From a rapid consultation of the scientific literature we discovered the interesting investigations of Bostwick [4] with a commercial substitute of formalin (Stat Fix). From the published components, we were able to prepare a mixture of PolyethylenGlycol (PEG), acetic Acid, Glycerol and ethyl alcohol (PAGA) in the lab, and this formulation was used with decent morphological results and no problems in Immunohistochemistry or Histochemistry. For almost one year PAGA was used in the substitution of formalin. Although PAGA was satisfactory as a routine fixative and not particularly expensive, an abrupt shortage of technical help led to a shift to a commercial alternative to formalin. No-Tox (EarthSafe Industries, Inc., Belle Mead, NJ 08502), a new commercial substitute of formalin was introduced in the market and was available in Italy. Unfortunately some difficulties were encountered with Immunohistochemistry (particularly Mib-1) and no suggestions were available from the producer in order to resolve this staining problem, and antigen retrieval experience was still limited [28,36]. We tested another new substitute of formalin, produced and marketed by Merck (Neo-Fix), which was an alcohol-based fixative (50% ethyl alcohol) and PEG. Since tissue shrinking was still acceptable and Mib-1 was easily detected, we shifted to the use of Neo-Fix and used it for more than 6 years, but due to commercial problems this fixative was no longer commercially available in Italy at the end of 2003. At that time we tested the commercially available alternatives to formalin present in the Italian market and selected FineFixx (Milestone, Italy) as the most suitable. FineFixx is an isopropyl and ethylic alcohol-based fixative (70% ethyl alcohol and tensioactive agents). The high alcohol percentage has evident shrinking artifacts but, despite this, morphology is acceptable. Immunohistochemistry and Histochemistry were also satisfactory.

In the quest for formaldehyde abolition, we also faced the problem of finding an effective substitute for the formaldehyde-formic acid mixture for a de-calcifying formula suitable for large orthopedic specimens. We obtained the recipe of de-calcifying fluid that was used routinely at the Surgical Pathology Dept. of Pini Orthopedic Hospital of Milan (Italy) (courtesy of A. Parafioriti M.D.) This method, based on Formic and Hydrochloric acids, was as effective as the former and easy to prepare in the laboratory.

## Materials and methods

The study was reviewed and approved by the Institutional board of the Molecular Biotechnology Center of the University of Turin, Italy.

In the present study we compared different commercial or laboratory prepared fixatives to the standard fixation protocols used in the laboratory for routine histology (FineFixx - Carnoy – B5).

The commercially fixatives tested included Cell-Block (Bio-Optica, Italy), as a Glyoxale - Ethyl Alcohol fixative; RCL2, a French proprietary formula based on Acetic Acid, Ethyl alcohol and non-reducing Carbohydrates (Celbio, Italy). The non-proprietary fixatives were prepared in our laboratory and tested: Zinc-based (ZBF and Z7), PAGA and derivatives with or without added buffers.

More than 200 inclusions using strict fixation protocols were prepared from surgical pathology specimens in which fresh tissue in excess of the diagnostic needs and protocol suggestions were available, as well as some autopsies in which the delay was not exceeding 24–36 hours after death. The diagnostic material was fixed in the routinely-used fixative. In a few cases (mainly autopsies) formalin-fixed blocks were prepared using a commercial buffered formalin (Histoline, Italy).

Fresh tissues were sliced at the thickness of 5 mm using a grossing board with different depth-cutting possibilities (Bio-Optica, Italy).

Tissues were immediately immersed in the different fixatives and routinely processed the following day (for the samples prepared on Friday the action of the fixatives was increased to 72 hours and subsequent processing steps were started the following Monday). Adjacent slices were immersed in different fixatives avoiding necrotic areas in order to standardize the sampling procedures and allow a valid morphological comparison.

Fixation tissues were then transferred to 70° ethyl alcohol and processed with routine blocks in a Shandon Excelsior processor. The protocol of dehydration – clarification and paraffin immersion was tailored for non-formalin fixation, as already in use for FineFixx.

The commercially-available fixatives tested Cell-Block (Bio-Optica, Italy), RCL2 (Celbio, Italy), Neo-Fix (Merck, Italy), were obtained by the producers or sale representatives as trial samples, as far as commercially available. For homemade recipes, Zinc Fixatives (ZBF and Z7), PAGA and PAGA-T were prepared according to the scientific literature [4,30,31]. FineFixx (Milestone, Italy), routinely used as a general fixative, served as a comparison [23]. For neurosurgical specimens, Carnoy's fixative was utilized [37].

For tissue from post-mortem examinations, formalin-fixed samples were available for comparison.

### Staining

Routine H&E was performed on slides using an automatic histology stainer (Leica, Italy) and the standard staining protocol for Fine-Fix fixed tissue was used. Giemsa, trichromic stain and Alcian blue (pH 1 and pH 2,5) PAS were also performed.

### Tissue micro array

Tissue micro arrays (TMA) were prepared using 3DHistec TMA Master. Acceptor blocks were designed to include 68 holes of 1.5 mm, and punching was then accomplished from all tissues from available blocks. A total of 5 TMA were prepared and cut.

Slides were then used for a morphological comparison of the performance of the different fixatives on a reduced number of slides in order to limit batch variability of staining.

TMA was also used to sample selected blocks for RNA extraction: four 1 mm cores were punched out from each block and extracted according to a tested protocol in use.

### Morphological evaluation

H&E stained sections from all inclusions were evaluated for different aspects of tissue and cellular details. The use of TMA for morphological comparison abolished the normal variation of staining intensity due to fading over time (fortnight substitution).

For comparisons, corresponding tissues were used that were fixed with the non-formalin fixatives used for routine diagnostic work. Fine-Fix was the reference fixative for most sampled cases, but Carnoy and B5 fixed specimens also entered into the comparison with the challenged fixatives.

### Immunohistochemistry

Routine immunohistochemistry was performed on non-formalin fixed tissues utilizing a Ventana Benchmark automated stainer and pre-diluted primary antibodies from Ventana, where available and when suitable for non-formalin fixation. In general, antigen-retrieval protocols [37] were used, with or without enhancing or highly sensitivity reactive agents. In a few cases, antibodies from different producers were used with the Benchmark stainer, either because they were unavailable from Ventana, or for substituting pre-diluted antibodies that gave unsatisfactory results (e.g. Baf47 from BD Buccinasco, Italy and WT-1 from Thermo Scientific, Aalst Belgium).

We had no problems in tailoring automated Immunohistochemistry, having already worked with Fine-Fix, to give acceptable results on the fixatives used in the present study.

### RNA extraction

Ten selected blocks of the same specimen were sampled for RNA extraction using four 1 mm cores punched out from tissue blocks with TMA Master.

Tissue cores were de-paraffinized with two passages in xylene, transferred to absolute ethanol, followed by 90% ethanol and then 70% ethanol. After spinning for 20 minutes at 4°C at 14,000 rpm pellets were warmed for 30 minutes at 37 C° (or until all ethanol had evaporated). Then, 200 μl of Digestion Buffer 1X with β-Mercaptoethanol (0.2%, final concentration) and Proteinase K (6 mg/ml, final concentration) were added. Samples were incubated overnight at 55°C, with gentle mechanical shaking.

A volume of Phenol/Chloroform mixture (7:3 v/v) was added to each sample, and tubes were gently mixed by inversion and incubated for 20 minutes on ice. Samples were spun for 20 minutes at 4°C at 14,000 rpm, and supernatants were then transferred to clean tubes and this procedure was repeated twice [38].

Samples were subsequently transferred to new tubes containing 5 μl of glycogen (1 mg/ml), precipitated with 3 volumes of ethanol-LiCl, and incubated overnight at – 20°C.

The following day, samples were spun for 20 minutes at 4°C at 14,000 rpm, and supernatants were discarded.

Pellets were washed once with 70% ethanol, left to dry at room temperature, and finally resuspended in 25 μl of DEPC water.

RNA concentration was determined at 260 nm and 280 nm with a Thermo Scientific Nanodrop 2000 spectrophotometer (EuroClone, Italy).

RNA samples were also tested for their quality using the RNA 6000 LabChip kit (Agilent Technologies) following the manufacturer’s instructions, and RNA analysis was evaluated with the 2100 Bioanalyzer (Agilent Technologies). This instrument performs electrophoresis for each RNA sample and delivers an RNA integrity number (RIN).

## Results

**Figure 1:**
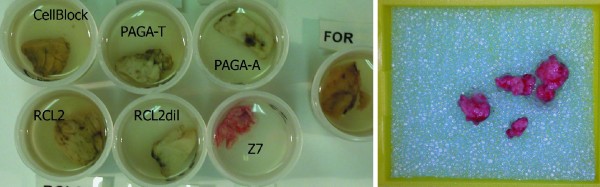
**Macroscopic aspect of fixation. **Tissue fragments still immersed in different fixatives after 16 hours (left); detail of Z7 fixed tissue following fixation, and within the biopsy cassette (right). Z7 tissue retains the original color of unfixed specimens while other samples show variations in color. It is important to be aware of and remember the difference of color modifications typical of each fixative, and not to only rely on discoloration to judge the final point of fixation. Some alcohol-based fixatives (like FineFixx) are second only to Z7 in the preservation of colors.

Macroscopical aspects of overnight fixation are shown in Figure 1. The action of each fixative modifies several physical characteristics of the samples in a unique way. Color and consistence are evident at a glance, while other features such as shrinkage and surface modifications are more subtle. In disagreement with a recent paper [39] that reported some difficulties in handling and sectioning of samples fixed in RCL2, we did not encounter any problems in these preparative aspects. The embedding process that follows fixation may largely modify the physical qualities of samples to be included in paraffin, and influence the ease in sectioning. As already stated, we used non-formalin fixatives for routine surgical specimen processing, so conditions were already set for non-formalin fixation.

A possible pitfall in processing may be over-dehydration related to over-timing of absolute alcohol exposure of tissues with less water content due to alcohol-based fixatives.

The high alcoholic concentration of some fixatives (FineFixx and RCL) may conflict with the automatic reagent turnover used in the Shandon - Thermo Excelsior tissue processor (Italscientifica, Italy) since it is based on dilution of alcoholic grade of the first dehydration bath and alcohol rotation.

In our experience, this possible technical pitfall is not clearly stated in the instruction manual of the instrument and may cause poor tissue processing due to delayed turnover and discarding of alcohol. A recommendation, when shifting from formalin to alcoholic substitutes, is to check the schedule of dehydrating agents in the tissue processor instrument and not to rely on alcoholometric-driven automatism since inaccurate dilution of alcohols will be detected.

It may be important to underscore that color preservation may be good enough to give the impression of inadequate fixation if discoloration is assumed to be a reliable sign of proper fixation. This phenomenon is present in many alcohol-based fixatives but is particularly striking with Z7, a mixture of Zinc salts.

In general, alcohol-based fixatives do not change color in the same way as formalin does. The modification of color may be generally used to control the process of fixation. Zinc salts as well as ethyl alcohol do not alter respiratory enzymes and oxygen-carrier proteins like hemoglobin and myoglobin which means that color fading (as induced by formalin action on tissues) is not induced by these chemical compounds and, especially with Z7, perfectly fixed tissue specimens preserve their original colors (Figure 1 right). Not being accustomed to these different macroscopic modifications of fixatives may induce the operator to interpret the lack of color modification as inadequate fixation and possibly prolong the fixation time.

**Figure 2:**
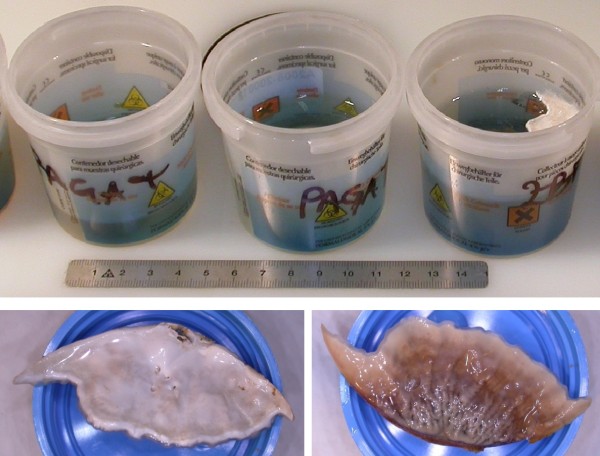
**Molds growing on the surface of fixative. **In a jar containing tissues fixed in ZBF one week after sampling a large colony of fungi is evident (top). Detail of upper and lower aspects of the colony (bottom).

An unusual phenomenon, only observed a few times, and just in the case of ZBF, was the development of molds on the top of the container (Figure 2). Although no apparent deterioration of the residual tissue was evident, this possible fungal growth on the surface of the fixative is undesirable.

**Figure 3:**
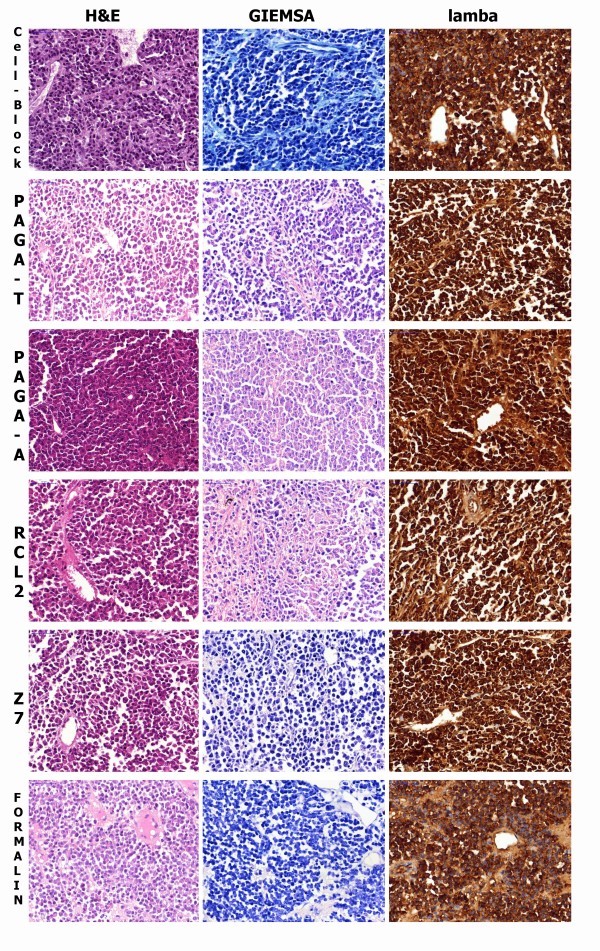
**Histological sections of a case of Myeloma. **Tissue fixed with Cell-Block, PAGA-T, PAGA-A, RECL2, Z7 and Formalin. Differences in tissue coarctation and nuclear details, as well as color affinity, are evident upon Haematoxylin- Eosin (H&E) and Giemsa (1stand 2 nd column) staining. Although formalin fixation shows less tissue shrinkage in comparison to alcoholic fixatives, along with a lower affinity to eosin, nuclear details are less well-preserved. In sections stained with Giemsa, Cell-Block, Formalin and Z7 are bluish in color and other fixatives tend to be pinkish. Giemsa is a pH-sensitive stain with a predominant blue hue with basic or neutral pH which turns to pink with a lower pH, as with RCL2 and PAGA that contain Acetic Acid at different concentrations. Nuclear details (as nucleoli) are sharper in formalin substitutes. Also the Golgi apparatus is more evident as paranuclear “hof”. Immunoperoxidase staining for lambda light chain (3rd column): strong positivity is present on all samples with some variation in cellular details and background. Immunostaining with automated procedure set for formalin-fixed tissues.

Morphology: Dye affinity alcohol-based fixatives, as well as Zinc-based ones, convey a higher affinity for dyes to the sections, especially Eosin, in comparison with neutral-buffered formalin or Glyoxale-based formulas. Nuclear structure is better preserved in alcohol-based fixatives. Conversely, shrinking artifacts are evident in alcoholic fixatives, and the entity of shrinkage is more evident when the alcohol concentration is higher than 50%. Also, Zinc-based formulas showed evident shrinkage of tissues (Figure 3).

For Immunohistochemistry (Figures 3 third column, 4, and 5), we had the advantage of utilizing routine staining protocols of an automated immunostainer (Ventana Benchmark - Diapath, Italy) already tailored for samples fixed with FineFixx (for surgical pathology antigens) or Carnoy Fixative for neuropathology (GFAP, Synaptophysin, Neu-N, HuC/HuD, Pituitary hormones etc.).

**Figure 4:**
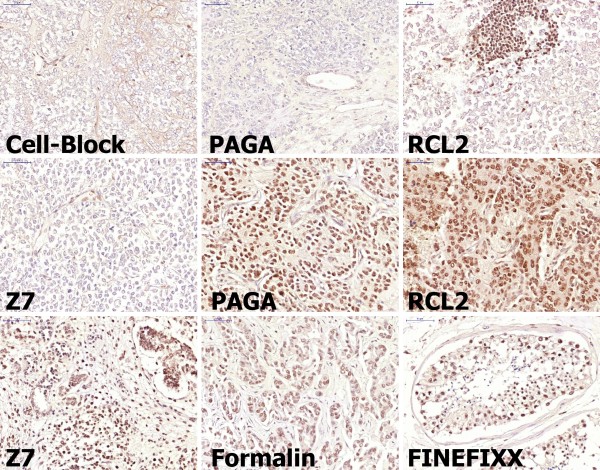
**Immunostaining for Baf47 nuclear antigen. **As expected no nuclear positivity is present in samples of a Rhabdoid Tumor of the kidney. Only endothelial nuclei are positive in all samples. Positive infiltrating lymphocytes are present and highlighted in the RCL2 sample. Other tumor samples such as Neuroblastoma (PAGA and RCL2, second row) Wilm's tumor (Z7) and ductal carcinoma (Formalin) as well as normal testis (FineFixx) show uniform nuclear positivity. [Manual antigen retrieval with microwave treatment in EDTA buffer pH 8.5.].

**Figure 5:**
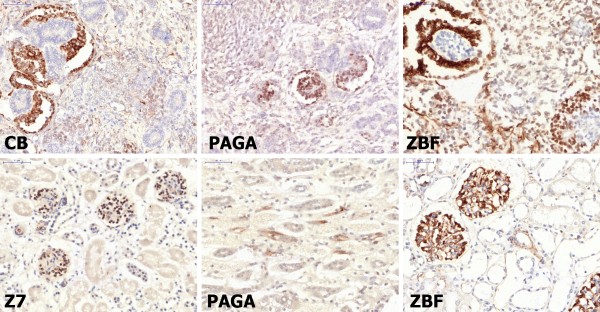
**Immunostaining for WT-1. **In Wilm's tumor (Cell-Block, PAGA and ZBF) staining is more intense in glomerular-like structures and in glomeruli of normal kidney (Z7 and ZBF). Some tubular cells are positive in the medulla only in PAGA-fixed tissue. [Manual antigen retrieval with microwave treatment in EDTA buffer pH 8.5.].

Nucleic Acid Extraction: as already reported in the scientific literature alcohol-based fixatives, as well as most of the proprietary formulas, are superior to formalin with regard to quality and quantity of Nucleic acid extractable from paraffin blocks. Similarly to RCL2, Cell Block (a glyoxale-based fixative) and PAGA-T were also superior to formalin in RNA preservation.

We focused on RNA extraction – an essential step for retro-transcription - due to higher sensitivity to denaturation and easy decay of this nucleic acid. Ribonucleic acid extraction confirmed that most of the fixatives tested were comparable or superior to formalin as far as quantity of RNA extracted was concerned. In Table 1 and Figure 3 the quantitative and qualitative results of RNA extraction are shown.

**Table 1:** Formulations of alternative fixatives discussed in study

	**Cell-Block**	**RCL-2**	**ZBF**	**Z7**	**PAGA-T**	**Fine-Fixx**
Ethyl Alcohol	50	62,5	-	-	56	70
Glyoxale	+	-	-	-	-	-
Acetic acid	-	5,00%	-	-	2,50%	-
Zn Cloride	-	-	0,50%	0,50%	-	-
Zn acetate	-	-	0,50%	-	-	-
Zn trifluoroacetate				0,50%		
Glycerol	-	-	-	-	4,00%	-
PEG	-	-	-	-	20,00%	-
Trealose	-	+	-	-	-	-
wetting agents	-	-	-	-	-	+
buffer	-	-	Tris–HCl	Tris–HCl	Tris–HCl	-
undisclosed	+	?	-	-	-	?
formaldehyde	-	-	-	-	-	-

**Figure 6:**
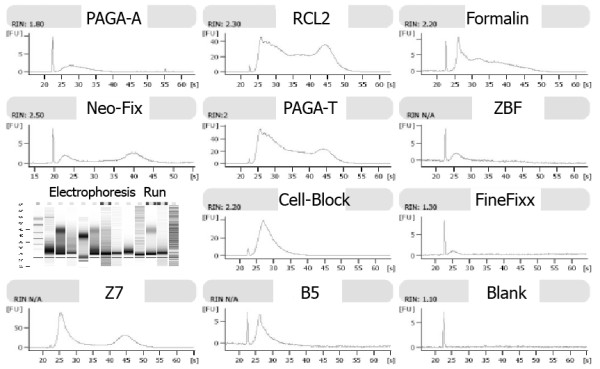
**Results of RNA 6000 LabChip analysis. **RNAs extracted from different fixatives show different RIN and electrophoresis curves.

The qualitative analysis of RNA by RNA Chip (Figure 6) showed RIN values spanning from 1.80 of PAGA-A to 2.50 of Neo-Fix, with CellBlock and formalin with a RIN of 2.2 and RCL2 of 2.30.

The striking difference of performance between PAGA-T (PAGA with buffer) and PAGA-A (with greater percentage of acetic acid) is interesting because it underscores the fact that a small difference in composition may influence the preservation of Nucleic Acids, while morphology itself is unaffected.

All fixatives tested permitted Q-RT-PCR to be performed for the 3 genes - beta2microglobulin, ribosomal protein S14 and glyceraldehyde phosphate dehydrogenase (GAPDH) - of increasing length of transcripts (data not shown).

Although these aspects of tissue integrity at the molecular level may be crucial for the application of molecular biology techniques for large quantities of surgical pathology specimens, other aspects of tissue handling may be relevant for morphological evaluation and easy tissue handling. The lag of time from sampling to proper fixation should be taken in account as well as the processing steps prior to paraffin inclusion [29]. Large variability of chemicals used in the processing steps, timing and temperature settings may have a non-marginal influence on the conservation of molecular constituents of tissues, and should be investigated and possibly standardized as far as possible. However, it appears to be difficult to find and standardize fixation and processing protocols suitable for routine surgical practices, which are thus refined to preserve tissue constituents as well as “snap freezing” procedures. The use of the HOPE technique is a promising alternative to bio-banking but not suitable for routine use [40]. The use of microwaves in tissue handling is very promising but may be not practical for busy labs unless dedicated instruments are available.

## Discussion

The key problem of fixation without formaldehyde is represented by the modifications of the tissues that alter their morphological aspect, when compared to what is routinely considered their standard counterparts. Any method of fixation or general handling of biological tissue for microscopic evaluation will introduce some modifications in the composition of the tissues that will alter their morphological aspect, and the formalin standard is quite variable from laboratory to laboratory as an average distribution.

Formalin, or more precisely buffered-formalin, follows the same rules but there is a general agreement that these artefacts are in some way standardized and acceptable as a “gold standard”. This widespread opinion has been solid enough to be interpreted as a “dogma”, in that this has become an opinion without a solid scientific background.

The enthusiasm for this new, highly versatile molecule, which allowed the use of a single fixative for different histochemical stains developed with dedicated fixatives was certainly justified in the two last centuries [41]. However, modern pathology relies on a wealth of new, sophisticated and predictive tools that may by applied on properly fixed tissues with different formulations of fixatives including formaldehyde-free ones.

One important point of this opinion that holds true is the necessity of standardization of the overall tissue processing steps, from the time gap from surgery to the staining, in order to homogenize the diagnostic evaluation and define a uniform diagnostic criteria.

The weakness of the “formalin dogma” is the fact that from many points of view formalin is not the best fixative but simply the most utilized. One aspect is the quality of microscopic slides and the accuracy and care of the fixation process: the limited penetration speed of fixatives should become an important intrinsic property of every fixative, and positive actions must be performed in order to permit adequate fixation in large and complex surgical specimens. However a somewhat fatalistic approach to the fixation process is the strongest motivation for formalin use, that is: when the specimen is immersed in an appropriate amount of formalin, the most important step of fixation is thus fulfilled.

Another aspect to be considered is the fact that formalin is actually a class of formaldehyde-based fixatives with a final aldehyde concentration of 4%. The most commonly used and recommended recipe is neutral-buffered formalin that is a 4% solution with the addition of a buffer (generally phosphate), to give a stable final pH close to neutrality. Different formulations are used under the general name of formalin, so the general agreement on “formalin fixation” is a somewhat very large and generic method encompassing quite different fixation conditions and practices. Moreover, the ready–to-use, commercially available, neutral-buffered formalin may not be equivalent or have standardized formulas, since some modifications of the composition are introduced by manufacturers in order to obtain a long-lasting product, suitable for use in laboratories for months or even years after production. Conversely, creating homemade recipes from 40% formaldehyde and tap or distilled water plus buffer may be time-consuming in preparation but may be more consistent over time, and freshly prepared without the necessity of stabilizing agents to ensure a long “shelf-life”.

One important issue to be considered is the determination of predictive or prognostic factors for targeted therapy of tumors and the necessity of reliable data that does not rely on the choice of fixative. The methylation state of O(6)-methylguanine-DNA methyltransferase (MGMT) in gliomas is a validated predictive response factor to chemotherapy. The report on the use of RCL2 in formalin substitution has demonstrated better performance of this alternative fixative in comparison to formalin [24]. The same applies in the comparison of Carnoy or FineFixx gliomas with formalin-fixed tumors. The peculiarity of a Pediatric Pathology Unit hampered the possibility of recruiting breast samples in this study for investigating the performance of the alternative fixatives in that particular context. We did however have the opportunity to study Her-2 expression in samples of breast carcinoma fixed in FineFixx. With adequate controls of positive breast tumors fixed in parallel in buffered formalin and Fine-Fixx and the validated immunistochemistry (Ventana, Martinengo,Italy) we obtained comparable results. ASCO-CAP guidelines [42] favor formalin fixation but do not exclude the use of different fixatives, recommending careful comparison and tight adherence to procedures and their validation for achieving proper and consistent results not influenced by fixation procedure. Even when formalin is used, the time of fixation should be tightly controlled (permitted range 6 – 48 hours). Accurate selection of the most suitable antibody from the validated ones, and the use of adequate controls may also permit the correct determination and interpretation of Herb-2 determinations [43] with alternative fixation formulas. As pointed out by oncologists, FISH determinations are less sensitive to fixation variables and its results are more relevant to biological responses of tumors to anti-Herb2 treatments [44]. The better preservation of nucleic acids obtained with alternative fixatives should be an ulterior point in favor of formaldehyde substitution as the primary fixative, as well as in the determination of molecular prognostic or predictive factors with in situ hybridization.

Recently, an innovative processing instrumentation has been proposed for large pathology departments. The system developed in Japan is based on microwave treatment and the proprietary formalin substitute (UMFIX = universal molecular fixative - Sakura Finetek USA, Inc.): a mixture of methanol and polyethylene glycol at a predetermined ratio [45]. In this way, an innovation introduced by industry as part of a cutting-edge processing procedure is undermining the “formalin dogma” with apparent no relevant opposition.

As a corollary to the introduction of UMFIX, it is also interesting to note that this fixative is not freely available on the market, but only sold to users of the system as a dedicated product. In fact we were intent on using UMFIX in our study, but were unable to obtain it from the national representative of the producer in Italy.

Another point to be considered is the use of formaldehyde in developing countries in which aspiration devices (hoods and aspirated cabinets) are rarely used and the disposal of toxic waste may also be inexistent or problematic [31].

The use of alimentary grade compounds for fixation (as is the case of some of the “homemade” substitutes) may be of benefit in these laboratories and be economically sustainable even in low income environments.

These are ideal times for a general effort in finding new more suitable fixatives that are less harmful, not only for morphological and immunohistochemical evaluation but, on the same note, for the preservation of important tissue components, allowing the widespread use of new technologies for diagnostic or scientific aims. A widespread effort towards practical scientific progress may improve the working environment of grossing and autopsy rooms, reduce or abolish the use of carcinogenic compounds, and be a concrete step towards more controlled processing procedures. Many of the commercially-available formalin substitutes are suitable for use in diagnostic purposes in routine histological practices. Conversely to the general opinion of the positive effects of free market, the use of proprietary formulas has been a real obstacle to generalized formalin substitution, as well as creating a higher cost of formalin substitutes. The possibility of preparing several different fixatives in the laboratory, and testing them with different tissues and working conditions should be underscored as a practical alternative to commercial products. This also overcomes the limited availability of some industrial products outside the USA, as well as the difficulties and relevant costs of transport and storage of highly flammable liquids.

The option of using a few different fixatives depending on specific pathological sectors may also be a positive step towards abolishing or significantly decreasing the use of formaldehyde.

Zinc-based fixatives, alcohol-tensioactive based and formulas including acetic acid and alcohol as important components, or glyoxale +/− alcohol appear to be suitable for general fixation, and may be effective alternatives to formalin as commercial products or laboratory-made reagents.

The conservation of important molecular tissue components appears to be superior in new fixatives when compared to formalin. The goal of a fixative suitable for routine diagnostic procedures, but that also preserves RNA, DNA and proteins, as well as in snap-frozen tissue samples, may be difficult to achieve immediately. However, the selection of less drastic fixatives, and the improvement and standardization of processing procedures, may preserve nucleic acids, and possibly proteins in order to integrate new techniques to routine surgical pathology determinations. Integrity of delicate tissue components may also be improved by combining new fixatives and physical agents like cooling and vacuum treatments when the time delay from theater to pathology laboratory may be critical. Microwave treatment is another possible processing method, but frozen tissue still remains more suitable for selected fields like proteomics.

## Conclusion

Our study demonstrates that several alternative fixatives are available as patented industrial products or as reagents easily prepared in the laboratory from chemicals present in the market. These fixatives are suitable for routine use for surgical pathology diagnostic work.

Although morphology may show fixation artefacts different from that induced by formalin fixation no real thread to surgical pathology diagnoses may be taken in consideration. Conversely alternative fixatives perform equally o better than formalin in all ancillary techniques used by modern pathologists.

No rational reasons hamper the complete substitution of formaldehyde as primary fixative in surgical pathology and in the medical research using laboratory animals. Times are ripe for the abolition of a carcinogen to human beings from Pathology Departments and research laboratories.

## Abbreviations

CalEPA: California Environmental Protection Agency; RoC/NTP: Report on Carcinogens - National Toxicology Program; ASCO: American Society of Clinical Oncology; CAP: College of American Pathologists; PAGA: Polyethylenglycol ethyl Alcohol Glycerol Acetic acid; PAGA-T: PAGA with buffer; PAGA-A: PAGA with greater percentage of Acetic acid; EPA: U.S. Environmental Protection Agency; OSHA: U.S. Occupational Safety and Health Administration; PEL: Permissible exposure limits; TWA: Time-Weighted Average; STEL: Short Term Exposure Limit; IARC: International Agency for Research on Cancer; TMA: Tissue Micro Arrays; H&E: Haematoxylin-Eosin; RIN: RNA Integrity Number; GAPDH: Glyceraldehyde Phosphate Dehydrogenase; MGMT: O(6)-Methylguanine-DNA Methyltransferase; FISH: Fluorescent In Situ Hybridization; UMFIX: Universal Molecular Fixative.

## Competing interest

The authors declare they have no competing financial interests.

## Authors’ contributions

EG selected fresh tissue for sampling in theatre and proper fixation in alternative fixatives. AV refined the RNA extraction protocol from tissue blocks and determined OD of nucleic acids. EE performed analysis quality assessment of RNA and performed RT-PCR of samples. CZ coordinated laboratory experiments and project implementation including TMA design and prepared the manuscript. MF, as principal investigator, conceived and designed the whole study, sought funding support, performed morphological evaluation and took part in writing the manuscript. All authors read and approved the final manuscript.
